# Combined Effects of Calcium Addition and Thermal Processing on the Texture and In Vitro Digestibility of Starch and Protein of Black Beans (*Phaseolus vulgaris*)

**DOI:** 10.3390/foods10061368

**Published:** 2021-06-13

**Authors:** Marbie Alpos, Sze Ying Leong, Indrawati Oey

**Affiliations:** 1 Department of Food Science, University of Otago, Dunedin 9054, New Zealand; marbie.alpos@postgrad.otago.ac.nz (M.A.); sze.leong@otago.ac.nz (S.Y.L.); 2 Riddet Institute, Palmerston North 4442, New Zealand

**Keywords:** starch digestibility, protein digestibility, texture, black beans, calcium, legume, thermal processing

## Abstract

Legumes are typically soaked overnight to reduce antinutrients and then cooked prior to consumption. However, thermal processing can cause over-softening of legumes. This study aimed to determine the effect of calcium addition (0, 100, 300, and 500 ppm in the form of calcium chloride, CaCl_2_), starting from the overnight soaking step, in reducing the loss of firmness of black beans during thermal processing for up to 2 h. The impact of calcium addition on the in vitro starch and protein digestibility of cooked beans was also assessed. Two strategies of calcium addition were employed in this study: (Strategy 1/S1) beans were soaked and then cooked in the same CaCl_2_ solution, or (Strategy 2/S2) cooked in a freshly prepared CaCl_2_ solution after the calcium-containing soaking medium was discarded. Despite the texture degradation of black beans brought about by increasing the cooking time, texture profile analysis (TPA) revealed that their hardness, cohesiveness, springiness, chewiness, and resilience improved significantly (*p* < 0.05) with increasing calcium concentration. Interestingly, beans cooked for 2 h with 300 ppm CaCl_2_ shared similar hardness with beans cooked for 1 h without calcium addition. Starch and protein digestibility of calcium-treated beans generally improved with prolonged cooking. However, calcium-treated beans cooked for 1 h under S2 achieved a reduced texture loss and a lower starch digestibility than those beans treated in S1. A lower starch digestion could be desired as this reflects a slow rise in blood glucose levels. Findings from this result also showed that treating black beans with high level of CaCl_2_ (i.e., 500 ppm) was not necessary, otherwise this would limit protein digestibility of cooked black beans.

## 1. Introduction

Legumes are known to contain a high amount of slowly digestible and resistant starch. This results in low postprandial glucose and insulin response when legumes are consumed by humans [1]. Legumes such as black beans are considered a low glycaemic index food, beneficial for preventing and managing type 2 diabetes [2]. The digestibility of starch is dependent on numerous factors, such as the starch structure, degree of crystallinity, amylopectin/amylose ratio, processing prior to digestion, and non-starch components such as protein, lipids, and antinutrients [3]. In particular, the cell structure of legumes has been found to greatly influence the hydrolysis of starch since some of the starch granules are entrapped in cell walls [4]. This serves as a barrier for digestive enzymes such as α-amylase to hydrolyse starch [5]. Moreover, the protein in legumes is broken down into amino acids by enzymes such as pepsin during digestion and plays a vital role in metabolism, muscle building and repair, organ function, and immune response [6]. It is bioencapsulated by a cell wall in the same cellular matrix as starch, which limits their accessibility to digestive enzymes and, subsequently, lowers their digestion and absorption in the human gastrointestinal tract [4,7,8].

Legumes are typically processed by soaking and cooking to modulate the bio-accessibility of nutrients and to eliminate or reduce antinutrients that limit their digestion [9]. Thermal processing can cause starch to undergo gelatinisation and swelling, denature protein, disrupt structure, and solubilise polysaccharides [10]. Disrupting the cell wall matrix of legume seeds by heat application can improve the digestibility of starch and protein by increasing their exposure and making them susceptible to enzyme hydrolysis [11]. Various studies have shown the effectiveness of food processing techniques in improving the starch and protein digestibility of various legume species by the elimination or reduction of antinutrients and modulating cell wall permeability, thereby increasing the exposure of starch and protein to enzymatic hydrolysis [12,13,14,15]. For instance, cooking by boiling in distilled water for 30 min improved the starch and protein digestibility and drastically decreased the amount of antinutrients such as tannin, phytic acid, trypsin inhibitor, and lectin in common beans, pea, and lentil [13]. Soaked and de-hulled kidney and faba beans also demonstrated a higher nutrient digestibility and loss of antinutrients than unprocessed samples [16]. Varying the cooking medium by addition of salt solutions could also potentially affect the nutrient digestibility of legumes [17]. For instance, addition of sodium bicarbonate was shown to enhance the rate of starch digestion in moth bean and horse gram [18]. Sodium salts are known to reduce antinutrients of legumes better than when cooking in water alone, thereby enhancing the bio-accessibility of nutrients to digestive enzymes [19,20]. However, the digestibility of protein in cowpeas was not affected by the type of cooking medium [13]. 

Texture is an important characteristic in food as it largely influences consumer acceptability [21]. While thermal processing is very effective in improving the degree of digestibility of nutrients, this technique can cause the softening of plant tissues such as in legumes by disrupting the cell wall components [22]. One of the common approaches known to help improve firmness in fruits and vegetables is the addition of calcium [23,24,25]. Calcium has been used in the canned legumes industry (up to 0.025 M or 1000 ppm concentration) since the 1980s to prevent legumes from losing firmness and becoming too mushy in order to meet consumer demand [26]. Calcium chloride (CaCl_2_) has been found effective in reducing the splitting and clumping of canned beans [25,27]. Calcium is usually added during the hydration or soaking of legumes to allow the uptake of calcium ion (Ca^2+^). Then, when beans are thermally processed, the exogenous Ca^2+^ will form complexes with demethylated pectin in the middle lamella of the cell wall due to the activation of pectinmethylesterase (PME) [28]. Heat treatment activates the enzyme PME, which de-methoxylates pectin, and thereafter crosslinks with calcium, strengthening the middle lamella in the cell wall and enhancing cell-to-cell adhesion [27,29]. Although calcium has been proven to prevent the loss of firmness in plant tissues, its consequence on the nutritional quality of cooked legumes is not yet fully understood. Legumes with firmer texture due to calcium addition may present a challenge for human digestive enzymes to access the nutrients upon consumption. There is a vast range of studies available in the literature to investigate the effect of calcium addition on the texture of cooked and canned legumes [25,27,29,30,31]. However, to date, there is very little information and understanding on its consequence on the starch and protein digestibility of cooked black beans.

The main aim of this study was to determine the combined effects of thermal processing and the addition of calcium on the in vitro starch and protein digestibility of black beans (*Phaseolus vulgaris*). Specifically, this study aimed to determine the effect of increasing cooking times (0, 1, and 2 h) and CaCl_2_ concentrations (0, 100, 300, and 500 ppm) on the textural properties, gelatinisation properties, and in vitro starch and protein digestibility of black beans. In this study, two strategies were adopted to prepare black beans: they were either soaked overnight to reduce the antinutrients and then cooked in the same CaCl_2_ solution (Strategy 1) or were cooked in a freshly prepared CaCl_2_ solution after the calcium-containing soaking medium (after overnight soaking with black beans) was discarded (Strategy 2). This would provide insight on the fate of the soaking water, whether to discard it prior to cooking or not. Information on the consequence of both strategies on the texture and in vitro starch and protein digestibility of cooked black beans was elucidated and compared.

## 2. Materials and Methods

### 2.1. Raw Material

A batch of 10 kg of black beans (dried) were purchased from a local store in Dunedin, New Zealand. Beans were sorted and those with physical damage and discoloration were discarded. Undamaged beans were vacuum-packed and stored at 4 °C until use.

### 2.2. Calcium Uptake during Soaking and Thermal Processing of Black Beans

The black bean samples (about 40 g per experimental variable) were soaked in different concentrations (0, 100, 300, and 500 ppm) of calcium chloride (CaCl_2_, Hawkins Watts, Auckland, New Zealand) at a 1:10 (*w*/*v*) seed to CaCl_2_ solution ratio for 24 h. After soaking, the samples were divided into two groups (approximately 20 g per glass bottles (Fisherbrand™, Pittsburgh, Pennsylvania)): cooked in the existing calcium chloride solution (hereafter referred to as “Strategy 1”), or calcium chloride solution was discarded and then the soaked beans were cooked in freshly prepared calcium chloride solution with the same concentration (0, 100, 300, and 500 ppm, at a 1:10 (*w*/*v*) seed to calcium-containing solution ratio) as they were soaked (hereafter referred to as “Strategy 2”). Then, the glass bottles with samples were placed in a stainless-steel pot pre-filled with boiling water. The beans were subjected to thermal processing using an electric coil cooktop (Westinghouse Neptune, 12.1 kW, set at high temperature) by boiling (>95 °C) for 0, 1, and 2 h. The temperature was constantly monitored using a thermocouple (Type K, 0.2 mm, Labfacility, West Sussex, UK) connected to a data logger (Pico TC-08, Pico Technology, St Neots, UK). After cooking, the beans were immediately cooled in an ice water bath (<4 °C) for at least 20 min. Each soaking strategy, CaCl_2_ concentration, and thermal processing combination was performed independently at least three times to be used for further analyses.

### 2.3. Microstructural Evaluation of Cooked Black Beans

To determine the microstructure changes that occurred in black beans after cooking, samples were lyophilised (FreeZone freeze dryer, Labconco, Kansas City, MO, USA) and then observed under a light microscope, as described by Gwala et al. [32]. Firstly, 0.6 mL of distilled water was added to the lyophilised powder (0.05 g), followed by addition of 20 µL of 0.1 N iodine to stain starch granules. Then, about 20 µL of stained sample was transferred on a microscope slide and examined under a light microscope (Ceti, Auckland, New Zealand) using the objective of 10× magnification. Micrographs were captured using a camera (Medline Scientific, Oxfordshire, UK) attached to the microscope and a camera control software (ToupTek ToupView, Zhejiang, China).

### 2.4. Texture Profile Analysis (TPA) of CaCl_2_-Soaked and Cooked Black Beans

**Figure 1:**
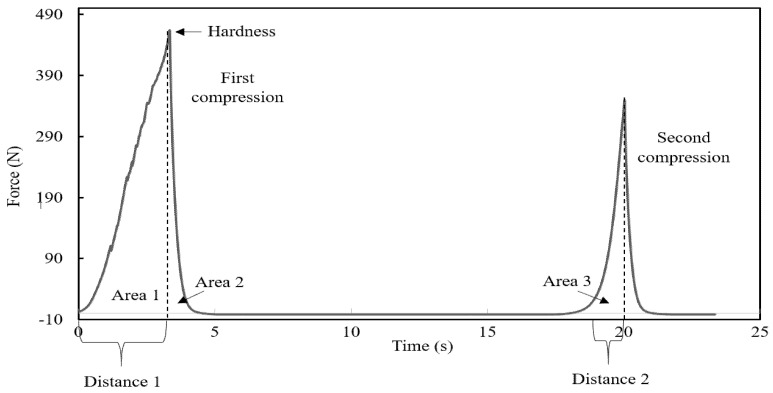
An example of the TPA curve of uncooked black beans determined in the present study.

In this study, the TPA analysis of Wani et al. [33] on kidney beans was adopted with modifications. Double compression or ‘two-bite’ tests on black bean samples, soaked in different calcium chloride solutions (0, 100, 300, and 500 ppm) and cooked in either existing (“Strategy 1”) or freshly prepared calcium chloride solution (“Strategy 2”) for 0, 1, and 2 h, were conducted by compressing at 50% strain (5 beans per compression, at its natural rest position with the hilum on its side) in a texture analyser (TA-HD plus, Micro Stable System, Surrey, UK) using a 50 mm diameter cylinder probe attached in a 250 kg load cell with a test speed of 1 mm/s. The textural properties measured from the texture profile analysis (TPA) curve (Figure 1) were hardness (peak force during the first compression, Newton), cohesiveness, springiness, chewiness, and resilience. The latter four texture parameters were derived from calculations using Equations (1)–(4), respectively [34]. The average of 10 compressions per treatment (i.e., 5 beans per compression, thus 50 beans in total) was reported.

Cohesiveness = Area 3/Area 1(1)

Springiness (mm) = Distance 2/Distance 1(2)

Chewiness (J) = Hardness × (Distance 2/Distance 1) × (Area 3/Area 1)(3)

Resilience = Area 2/Area 1(4)

where Area 1 is the downstroke energy of the first compression, Area 2 is the upstroke energy of the first compression, Area 3 is the downstroke energy of the second compression, Distance 1 is the original compression distance, and Distance 2 is the distance of the detected height during the second compression [35]. 

### 2.5. Determination of Gelatinisation Properties of CaCl_2_-Soaked and Cooked Black Beans Using Differential Scanning Calorimetry (DSC)

Gelatinisation parameters of black beans, as influenced by addition of calcium and thermal processing, were measured using DSC Q2000 (TA Instruments, New Castle, DE) according to the reported procedure of Chigwedere et al. [36]. Black bean samples were prepared by freeze-drying and milling to pass through a 425 µm mesh screen. Then, 3 mg of the lyophilised sample was weighed in a hermetic aluminium pan (TA Instruments Tzero, New Castle, DE) and 15 µL of Milli-Q water was added using a micro-syringe. The pan was sealed, reweighed, and allowed to equilibrate at room temperature for 24 h to evenly disperse the water into the sample. The sealed sample pan was then placed in the DSC chamber together with an empty pan as a reference and heated from 20 to 120 °C, at 5 °C/min. Transition temperatures (T_o_: onset, T_p_: peak, and T_c_: conclusion) and gelatinisation enthalpies (∆*H*) were generated from the endothermic curves obtained using the Universal Analysis 2000 software (Version 4.5A, TA Instruments-Waters LLC, Shanghai, China). The DSC measurement was repeated twice for each sample.

### 2.6. Determination of Total Starch Content of CaCl_2_-Soaked and Cooked Black Beans

Total starch (TS) measurement, based on the total starch assay kit (AA/AMG) from Megazyme (Wicklow, Ireland), was conducted to determine the amount of beans needed for the digestibility assay. The result was used later as TS content in undigested samples to calculate the starch hydrolysis (%) in the digest (refer to Section 2.8.1). Black beans soaked and cooked in existing or freshly prepared calcium chloride solutions at different concentrations (0, 100, 300, and 500 ppm) for 0, 1, and 2 h were freeze-dried and then milled to pass through a 425 µm mesh screen before analysis. 

Ground black bean samples (20 mg) were placed in 50 mL centrifuge tubes, and 5 mL of 100 mM sodium acetate buffer (pH 5) were added to each tube. They were vortex mixed for 5 s, and 50 µL of thermostable α-amylase (from *Bacillus* sp., 3000 U/mL, Megazyme) was added to convert the starch to maltodextrins. After vortex mixing again for 3 s, the samples were transferred to a boiling water bath (Grant, Cambridge, England) set at 100 °C to solubilise starch. They were vortex mixed every 5 min for 15 min. Immediately after, the tubes were transferred to a water bath (Grant, Cambridge, England) at 50 °C for 5 min. Then, 50 µL of amyloglucosidase (from *Aspergillus niger*, 3260 U/mL, Megazyme), which hydrolyses the maltodextrin to D-glucose, was mixed into each tube. The tubes were then heated in a water bath (Grant, Cambridge, England) at 50 °C for 60 min without further mixing. The mixtures were topped up to 50 mL with 100 mM sodium acetate buffer (pH 5). Then, the samples were centrifuged at 2056 g (Beckman GPR Centrifuge, Brea, CA, USA) for 10 min. Fifty microliters of each sample, glucose control (1 mg/mL in 0.2% benzoic acid, Megazyme), and 10 mM sodium acetate buffer (pH 5, reagent blank) were added with 1.5 mL glucose oxidase/peroxidase (GOPOD) reagent (Megazyme), which were prepared in duplicate then heated in a water bath (Grant, Cambridge, England) at 50 °C for 20 min. Lastly, the absorbance of the samples was read at 510 nm against the reagent blank using a plate reader (BioTek^®^ Synergy™ 2, Winooski, VT, USA). The total starch was expressed as mg per g dry weight of sample, in which a conversion factor of 0.9 (=162/180) was used to account for free D-glucose conversion to anhydro D-glucose (as occurs in starch). The total starch content of each sample was corrected against the presence of free D-glucose by repeating the above-mentioned steps, however without the addition of α-amylase and amyloglucosidase enzymes prior to reaction with GOPOD reagent, according to the instructions from the Megazyme kit. Each sample was extracted and analysed in triplicate for total starch and free D-glucose. The mean values from three replicates were obtained. 

### 2.7. Simulated In Vitro Human Gastric Intestinal Digestion Assay on CaCl_2_-Soaked and Cooked Black Beans

Prior to the digestibility assay, each of the black bean samples (5 g)—soaked in different calcium chloride solutions (0, 100, 300, and 500 ppm) and cooked in either existing or freshly prepared calcium chloride solution for 0, 1, and 2 h—was compressed using the texture analyser (TA-HD plus, Micro Stable System, Surrey, UK) at 90% strain using a 50 mm cylinder probe attached in a 250 kg load cell. This compression mimics the human mastication of the beans before undergoing digestion [37]. The digestibility assay adopted in this study was a general standardised and practical static digestion method developed by Infogest [38]. This mimics in vivo digestion in terms of the digestive enzymes and their concentrations, pH, digestion time, and salt concentrations. Preliminary reproducibility experiments showed that the in vitro digestion assay generates highly reproducible results (data not shown) over same sample, with a <10% coefficient of variance. 

#### 2.7.1. Preparation of Digestion Solutions

All the solutions were prepared on the same day of the assay according to the work of Abduh et al. [39]. Saliva juice (hereafter referred to as “SJ”) was prepared by adding 0.117 g (2 mM) of sodium chloride, 0.149 g (2 mM) of potassium chloride, and 2.1 g (25 mM) of sodium bicarbonate in 1 L of Milli-Q water. Alpha amylase solution (hereafter referred to as “AA”) was prepared by adding 1.25 g of *Aspergillus oryzae* α-amylase (Sigma, 30 U/mg, St. Louis, MO, USA) in 100 mL of Milli-Q water. Gastric solution (hereafter referred to as “GS”) was prepared by mixing 8.8184 g (151 mM) sodium chloride and 2.1 g potassium chloride (28 mM) in 1 L 1 mM hydrochloric acid at pH 3. Then, 4 g porcine stomach pepsin (AppliChem A4289, 0.7 FIP-U/mg, Barcelona, Spain) was added in 100 mL of GS. For the small intestinal solution (hereafter referred to as “SIS”), 1 g of porcine pancreas pancreatin (Sigma P1750, 4 × USP Missouri, USA) and 0.8452 g of porcine bile extract (ChemCruz SC-214601, Dallas, TX, USA) were added into 100 mL of 0.1 M sodium bicarbonate at pH 7.

#### 2.7.2. In Vitro Digestibility Procedure

Five grams of compressed black beans were placed in 100 mL Schott glass vessels and added with 20 mL of SJ. These were incubated at 37 °C (Contherm Scientific Ltd., Hutt City, New Zealand) for 5 min with shaking (55 strokes/min) on a shaker with a rocking motion tilt angle of 7° (DLAB, SK-R1807-S, New Territories, Hong Kong). After that, 5 mL of AA was added and shook again for 5 min. Then, the solution was adjusted to pH 3 with 1 M HCl to deactivate amylase. Pepsin containing GJ solution (20 mL) was added to each digestion vessel and incubated at 37 °C for 120 min with shaking. After the 2 h gastric digestion, the pH was adjusted to 7 with 1 M NaOH to deactivate pepsin. Then, 40 mL of pancreatin/bile containing SIS solution was added and incubated again at 37 °C for 240 min with shaking. 

#### 2.7.3. Collection of Digests along the Gastrointestinal Digestion

For starch digestibility measurement, 0.5 mL of digesta were collected from the gastric phase (after addition of pepsin containing GJ solution) at 0 and 120 min and small intestinal phase (after addition of pancreatin/bile containing SIS solution) at 0, 20, 30, 40, 60, 90, and 120 min with immediate heat shock in a boiling water bath (>95 °C for at least 15 min, Techne FTE10ADC, Staffordshire, UK) to inactivate digestive enzymes [4]. Then, 2.5 mL of 100 mM sodium acetate buffer (pH 5) was added to the collected digesta and centrifuged at 2056× *g* (Beckman GPR Centrifuge, California, USA) for 20 min. The supernatant was kept at 4 °C until analysis (refer to Section 2.8.1) within 24 h.

Digesta (0.5 mL) for protein digestibility measurement were collected in Eppendorf tubes pre-filled with 0.5 mL of 20 (*v*/*v* %) TCA [40] at different time points (0, 30, 60, and 120 min) during the gastric phase (after addition of pepsin containing GJ solution) and at 0, 20, 30, 40, 60, 90, and 120 min in the small intestinal phase (after addition of pancreatin/bile containing SIS solution). Collected digesta were stored in a freezer (−18 °C) until measurement (refer to Section 2.8.2).

### 2.8. Measurement of Starch and Protein Digestibility

#### 2.8.1. Determination of Hydrolysed Starch for CaCl_2_-Soaked and Cooked Black Beans

The D-glucose assay kit (GOPOD format) from Megazyme (Wicklow, Ireland) was used for measuring starch digestibility, as previously employed by other studies [15,33,34]. The supernatants collected from the in vitro digestibility assay at different time points during gastric and small intestinal phase (see Section 2.7.3) were placed in 50 mL centrifuge tubes and added with 50 µL amyloglucosidase (from *Aspergillus niger*, 3260 U/mL, Megazyme). They were vortexed and heated in a water bath (Grant, Cambridge, England) at 50 °C for 60 min, with intermittent mixing in a vortex mixer. The tubes were then topped up to 5 mL with 100 mM sodium acetate buffer (pH 5). Then, the samples were centrifuged at 2056 *g* (Beckman GPR Centrifuge, Brea, CA, USA) for 10 min. Fifty microliters for each of the sample, glucose control (1 mg/mL in 0.2% benzoic acid, Megazyme), and 100 mM sodium acetate buffer (pH 5, reagent blank) were added with 1.5 mL GOPOD reagent. These were performed in duplicate, then heated in a water bath (Grant, Cambridge, UK) at 50 °C for 20 min. Lastly, the absorbance was read at 510 nm against the reagent blank using a plate reader (BioTek^®^ Synergy™ 2, Winooski, VT, USA). The amount of D-glucose in the sample digest was calculated from the absorbance and then the result was converted into hydrolysed starch in sample digest using 0.9 (=162/180) to account for free D-glucose conversion to anhydro D-glucose (as occurs in starch). Then, the hydrolysed starch (or starch hydrolysis %) in the digest at each sampling time point along the digestion was calculated by dividing the hydrolysed starch in sample digest with TS content in undigested sample, as described earlier in Section 2.6.

#### 2.8.2. Determination of Hydrolysed Protein for Ca-Soaked and Cooked Black Beans

The measurement of protein digestibility was performed using the *o*-phthaldialdehyde (OPA) assay, which determines the free α-amino groups of the peptide fractions [40]. The OPA reagent was prepared by mixing 50 mL of 100 mM sodium tetraborate, 10 mL of 10% (*w*/*v*) sodium dodecyl sulphate (SDS), 80 mg of OPA previously dissolved in 2 mL of methanol, and 200 µL of β-mercaptoethanol. The mixture was further diluted to 100 mL with Milli-Q water. The digesta collected from the in vitro digestibility assay at different time points during gastric and small intestinal phase (see Section 2.7.3) were centrifuged at 13,000 *g* (IEC Micromax, Needham Heights, MA, USA) for 5 min and were transferred into wells of a 96-well plate. Then, OPA reagent was added to each well using a multichannel pipette to achieve a ratio of 2:15, sample to OPA. Using the plate reader (BioTek^®^ Synergy™ 2, Winooski, VT, USA), the solutions were gently mixed for 30 s and allowed to react for another 90 s. Then, the absorbance was measured at 340 nm. The α-amino groups or amounts of peptides produced during pepsinolysis were calculated as *L*-serine equivalents with reference to the *L*-serine standard curve, at different concentrations (0, 25, 50, 100, 150, 200, 300, 400, and 500 mg/L).

The total α-amino groups in the undigested samples were also determined [41]. Five milligrams of lyophilised sample were hydrolysed with 1 mL of 6 N HCl at 110 °C for 24 h. A rotary evaporator (Buchi Rotavapor, Flawil, Switzerland) was used to remove the acid, after which the sample was diluted with 5 mL of Milli-Q water. Thereafter, the samples were measured using the OPA spectrophotometric assay, as described above. Protein hydrolysis % was calculated as shown in Equation (5):

(5) $$\mathrm{Protein}\;\mathrm{hydrolysis}\;(\%)=\frac{{\mathrm{NH}}_{2\;(\mathrm{digesta})}{\;-\mathrm{NH}}_{2\;(\mathrm{initial})}}{{\mathrm{NH}}_{2\;(\mathrm{total})}{\;-\mathrm{NH}}_{2\;(\mathrm{initial})}}\times 100$$

where NH_2 (digesta)_ is the *L*-serine equivalent at each digestion time point, NH_2 (initial)_ is the *L*-serine equivalent at the start of digestion (0 min sample at gastric phase), and NH_2 (total)_ is the *L*-serine equivalent of the undigested samples.

### 2.9. Statistical Data Analysis

Experimental data were reported as mean values with standard deviation. Data collected were analysed using Student’s *t*-test for single comparison and analysis of variance (ANOVA) with Tukey’s post hoc multiple comparison test at a 0.05 level of significance to determine significant differences in the measurements of black beans soaked at different calcium chloride solutions and cooked for 0, 1, and 2 h. Statistical analyses were executed using SPSS version 25 (IBM Corp., Armonk, NY, USA).

## 3. Results and Discussion

### 3.1. Microstructures of Black Beans as Affected by Increasing Thermal Processing Duration

**Figure 2:**
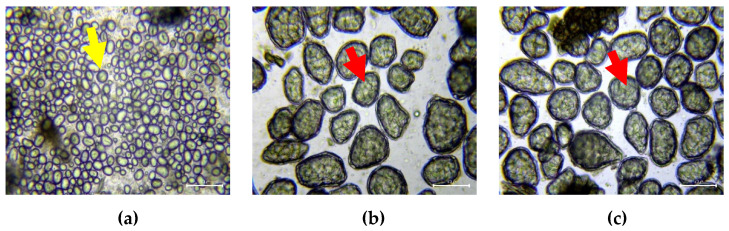
Light microscope images of parenchyma cells of uncooked (**a**) black beans cotyledon and those cooked (~100 °C) for (**b**) 1 and (**c**) 2 h stained with 0.1N iodine solution. The yellow arrow indicates the free starch granules while the red arrow indicates intact cells. Scale bar = 100 µm.

The microstructural behaviour and changes of the parenchyma cells of black beans cotyledon due to thermal processing were observed under a light microscope. The representative micrographs are shown in Figure 2 as a function of increasing cooking time (0, 1, and 2 h). For uncooked black beans, the abundant presence of free starch granules with round and oval shapes was observed without cell separation (Figure 2a). This microstructure phenomenon can be due to the strong middle lamellae which glues the cell together. Upon tissue disintegration, such as lyophilisation and milling, cell breakage occurs instead of cell separation, leading to the release of cell contents, including the starch granules [36]. This was similarly observed in uncooked legumes in previous works [36,42,43,44]. When black beans were cooked for 1 and 2 h (Figure 2b,c), starch swelled due to the application of heat, wherein starch became gelatinised, resulting in the rounding off of the cells with greater cell separation due to pectin solubilisation and middle lamellae weakening [22]. Despite being cooked and mechanically disintegrated by milling (during sample preparation), the parenchyma cells (diameter of 50–100 µm) of the cotyledon of black beans remained intact. The cell wall encapsulated and trapped the densely packed starch granules embedded in the proteinaceous matrix, as observed in the microscopic images of the cooked samples. Such morphology was similarly described by several authors in other legumes [1,8,32,45]. Overall, there was no visible change in the cell wall morphology (e.g., size and thickness) between the cooked (1 and 2 h) black beans. Based on the micrographs, it could be speculated that although starches were gelatinised after cooking, the presence of the intact cell wall protecting the gelatinised starch may limit the accessibility of starch and proteins towards digestive enzymes.

### 3.2. Effects of Increasing Calcium Concentration and Duration of Thermal Processing on the Textural Properties of Black Beans

Texture profile analysis (TPA) was conducted to determine the textural properties (hardness, cohesiveness, springiness, chewiness, and resilience) of soaked black beans cooked at 0, 1, and 2 h in calcium chloride (CaCl_2_) solutions (0, 100, 300, and 500 ppm) employing two calcium uptake strategies (Strategies 1 and 2). The results of these parameters are summarised in Table 1. The following sections discuss the effect of cooking time on black beans without the influence of added calcium. The combined effect of increasing calcium concentration and thermal processing duration is also presented.

#### 3.2.1. Effects of Increasing Cooking Time on the Texture of Black Beans without Receiving Calcium Treatment

As cooking time was increased up to 2 h, hardness, cohesiveness, springiness, chewiness, and resilience decreased significantly (*p* < 0.05) for black beans soaked and cooked without calcium (Table 1, 0 ppm CaCl_2_ values). Therefore, thermal processing clearly influenced the TPA parameters of black beans. Expectedly, uncooked beans were at least 14- and 21-fold harder and more structurally intact than 1 and 2 h cooked beans, respectively. This is the main reason legumes are normally cooked to have an acceptable texture by softening the seed coat and cotyledon. With respect to cohesiveness, soaked uncooked beans were generally more cohesive, up to 19%, compared to 2 h cooked beans, as the uncooked beans were harder and therefore able to withstand the deformation during compression. Uncooked black beans were observed to have significantly (*p* < 0.05) higher springiness (at least 41% higher) than any thermally treated beans, indicating higher recovery of bean height after first compression in the texture analyser. This is expected as the cooked beans’ structures are more compromised and softer, preventing the springiness phenomenon. Chewiness, which is the energy required to masticate the food to achieve a desired consistency for swallowing [34], was significantly (*p* < 0.05) higher (up to 59-fold higher) in uncooked compared to any cooked black beans. Expectedly, uncooked beans are harder and more structurally intact; therefore, more energy is needed to break them down. A similar finding was observed by Wani, Sogi, Wani, and Gill [33]. Lastly, resilience, which defines “how well the samples fight back to recover its original height” [35], significantly (*p* < 0.05) reduced, by at least 47%, for thermally treated black beans. Resilience differs from springiness such that it is expressed as a ratio of energies instead of distance. Resilience is a useful texture parameter because humans, who can easily sense the distance a food takes to regain its position, cannot determine the energy required to do so [46]. Since cooking the beans has softened their texture and reduced the distance they recovered during deformation, these explain a lower resilience observed for cooked samples in this study. Several authors have reported the same effect of cooking on the texture of other beans [47,48,49].

Based on Table 1 (refer to 0 ppm CaCl_2_ values), there was no difference observed between 1 and 2 h cooked beans in terms of their hardness, chewiness, and resilience. Hardness, which is an important texture parameter as it dictates the suitability of legumes’ consumption and consumer acceptability [50], was the same for 1 and 2 h cooked black beans. Therefore, cooking the black beans for up to 2 h was not necessary since similar hardness can be achieved with 1 h cooking. However, both cohesiveness and springiness showed a significant (*p* < 0.05) decrease when the thermal processing of black beans was prolonged to 2 h as compared to 1 h. This result suggested that beans that were cooked longer would be less resistant to compression during oral mastication by humans and do not have enough structural integrity to recover their height when destroyed. Moreover, the degree of texture degradation for uncooked and thermally treated beans for 1 and 2 h was not statistically significant (*p* > 0.05) between Strategy 1 and 2. Despite such similarity in texture properties for both strategies, various research has found that the soaking of legumes leads to the greatest leakage of antinutrients into the soaking water [13,16,51]. Hence, the removal of the soaking solution in Strategy 2 before cooking is expected to reduce the antinutritional compounds in legumes that limit the absorption of essential nutrients in the body.

#### 3.2.2. Effects of Calcium Addition during Soaking and Cooking on the Texture of Black Beans

Prior to cooking, the impact of increasing concentrations of CaCl_2_ (0, 100, 300, and 500 ppm) during soaking on the different texture parameters of soaked uncooked beans was found to be insignificant (Table 1, refer to 0 h cooking values). In other words, the infusion of calcium into the intact uncooked beans during 24 h soaking alone was almost negligible on their texture since uncooked beans were still intact and the seed structure was not disrupted as compared to cooked beans. When the CaCl_2_-treated black beans were cooked for up to 2 h, all texture parameters (hardness, cohesiveness, springiness, chewiness, and resilience) generally decreased with increasing cooking time (Table 1), except for the cohesiveness of any CaCl_2_-treated cooked beans under Strategy 2. Texture degradation can be due to the loss of membrane integrity with prolonged heating. However, it is important to note that the extent of texture degradation caused by prolonged cooking was significantly (*p* < 0.05) reduced due to CaCl_2_ treatment when compared to cooked beans that did not receive any CaCl_2_ treatment in advance (see Section 3.2.1). After cooking for 1 and 2 h, black beans became more permeable, causing an apparent increase in all texture parameters with increasing CaCl_2_ concentration (Table 1). As shown in a previous study, a significant firming effect of calcium was also observed in cooked carrots when compared to raw carrots [52,53]. 

Divalent cations such as calcium are known to strengthen the middle lamella in the cell wall of legumes by forming insoluble salt bridges with the free carboxyl groups in the pectin molecules [28]. The application of heat at 50–80 °C activated the enzyme pectin methylesterase (PME) that de-methoxylates pectin to promote the formation of insoluble calcium-pectin complex [29]. Further heating by boiling potentially enhanced the rate of calcium diffusion into the beans due to the increased membrane permeability. Smout et al. [54] has reported an improved firmness in carrots when calcium treatment is combined with heating than with calcium pre-treatment alone. Furthermore, an increase in firmness was observed by Wang, Chang, and Grafton [25] in canned beans.

The impact of increasing CaCl_2_ concentration on the hardness of cooked beans was found to differ for Strategy 1 and Strategy 2 (Table 1). For instance, a significant (*p* < 0.05) improvement in the hardness of cooked beans was observed when beans were soaked overnight followed by cooking for 1 h in 300 ppm CaCl_2_ under Strategy 1, while a lower dosage level of 100 ppm CaCl_2_ was found to be adequate in achieving a similar improvement in hardness (within the range of 46.82–48.04 N) for 1 h cooked beans under Strategy 2. The application of 500 ppm CaCl_2_ further increased the hardness of beans during the course of 1 h cooking under both strategies, but the cooked beans under Strategy 2 were found to be significantly (*p* < 0.05) harder (79 N in Strategy 2 vs. 67 N in Strategy 1). For black beans cooked for 2 h, it appeared that application of CaCl_2_ up to 300 ppm under Strategy 1 had no significant impact in preventing the beans from losing their hardness. It was only when the beans were soaked and cooked in 500 ppm CaCl_2_ under Strategy 1 that the hardness of the 2 h cooked beans significantly (*p* < 0.05) recovered by 62.9% to that of 2 h cooked beans in the absence of CaCl_2_. Under Strategy 2, CaCl_2_ treatment showed its effectiveness in preventing the 2 h cooked beans from losing their hardness significantly (*p* < 0.05), by 72.5% and 129%, compared to the 2 h cooked beans in 0 ppm CaCl_2_, when applied at 300 and 500 ppm, respectively. It appeared that replacing the CaCl_2_ solution prior to cooking, after soaking, in Strategy 2 could have enabled more calcium ions to diffuse into the beans during cooking, as evidenced by the significant increase in hardness at 300 and 500 ppm CaCl_2_. According to Fick’s diffusion law, diffusivity increases with temperature, resulting in a faster imbibition rate of legumes during cooking [55,56,57].

The cohesiveness of black beans was found to increase with increasing concentrations of CaCl_2_ when cooked for 1 and 2 h (Table 1). A higher concentration of CaCl_2_ (i.e., >300 ppm) appeared necessary to maintain the cohesiveness of 1 h cooked black beans in Strategy 1. Moreover, a CaCl_2_ treatment of only 100 ppm in Strategy 2 was needed to achieve the same cohesiveness (i.e., 0.25) as those beans treated with 500 ppm CaCl_2_ in Strategy 1 when cooked for 2 h. Hence, Strategy 2 was more effective in lowering the amount of calcium needed to preserve cohesiveness of black beans. This finding can be related to the hardness result wherein Strategy 2 led to production of harder beans than Strategy 1, possibly due to the greater formation of calcium-pectin crosslinks, making them more resistant to compression or oral mastication, and hence increased cohesiveness.

Springiness, or the distance when black beans spring back after compression, decreased with increasing cooking time, and was significantly (*p* < 0.05) degraded in 2 h cooked black beans (Table 1). The loss of structural integrity brought about by cooking lowered the ability of food samples to recover their height after deformation. However, the springiness of cooked beans was observed to increase with increasing CaCl_2_ concentrations (Table 1). The high amount of calcium could have helped to strengthen the tissue cell wall, and thus the cooked beans became more resistant to deformation and retained their structure even after compression. It is also observed that Strategy 2 appeared to preserve the springiness of cooked beans better than Strategy 1, where cooking black beans for 1 and 2 h employing Strategy 2 reduced the amount of CaCl_2_ required to make them as springy as samples in Strategy 1. For example, the same springiness (0.43–0.44 mm) was achieved when black beans were cooked for 1 h with 300 ppm CaCl_2_ in Strategy 1 and with 100 ppm CaCl_2_ in Strategy 2. 

Chewiness, or the energy required to masticate the beans, was found to decrease with increasing cooking time and increased with calcium concentration (Table 1). At 1 h of cooking, the chewiness of black beans cooked in all concentrations of CaCl_2_ was significantly (*p* < 0.05) higher than samples without added CaCl_2_ for both strategies. However, only the beans treated at higher CaCl_2_ concentrations showed such effect at 2 h cooking for both Strategies 1 (at 500 ppm CaCl_2_ only) and 2 (at both 300 and 500 ppm CaCl_2_). Cooked beans subjected to Strategy 2 consistently had a higher chewiness than those treated with Strategy 1, regardless of the CaCl_2_ concentrations and cooking duration. For instance, cooking for 1 h and the addition of 300 ppm CaCl_2_ in Strategy 2 increased the chewiness of black beans by 53% as compared to those samples treated with the same CaCl_2_ concentration in Strategy 1. 

Based on Table 1, it was also found that calcium addition did not affect the resilience of cooked black beans as much as the other texture parameters. Nevertheless, CaCl_2_ treatment at higher concentrations (300 and 500 ppm) was shown to increase the resilience (ratio of energy needed to regain their height after compression) of the cooked beans regardless of the cooking duration. For instance, at 1 h cooking, resilience increased by 25.17% and 24.22% in black beans cooked with 300 and 500 ppm CaCl_2_ respectively, when compared with the untreated samples (0 ppm CaCl_2_) in Strategy 1, while there was no difference between 1 h cooked samples with different CaCl_2_ concentrations in Strategy 2. For 2 h cooked beans, resilience improved significantly (*p* < 0.05) only when subjected to CaCl_2_ treatment of concentrations greater than 300 ppm under Strategy 2, when compared to their untreated counterpart (i.e., in absence of CaCl_2_). A significant improvement in the resilience of 2 h cooked beans with increasing CaCl_2_ concentrations was not observed under Strategy 1.

This study indicated that textural parameters of black beans increased with CaCl_2_ concentration. The addition of CaCl_2_ was able to minimise the loss of firmness, especially at higher concentrations (300 and 500 ppm). For instance, black beans cooked for 2 h with 300 ppm CaCl_2_ had the same hardness as samples cooked for 1 h without calcium. When CaCl_2_-treated samples (300 and 500 ppm) were cooked for only 1 h, the hardness values were more than twice the untreated samples (0 ppm CaCl_2_). Furthermore, Strategy 2 preserved the texture better than Strategy 1, revealing the importance of replacing the CaCl_2_-containing soaking water before cooking black beans in CaCl_2_ solutions. Preserving the texture of cooked legumes is important in preventing too much softening and reduce clumping and splitting when processing at the industrial scale.

### 3.3. Effects of Calcium Addition and Duration of Thermal Processing on Gelatinisation Properties of Black Beans

**Figure 3:**
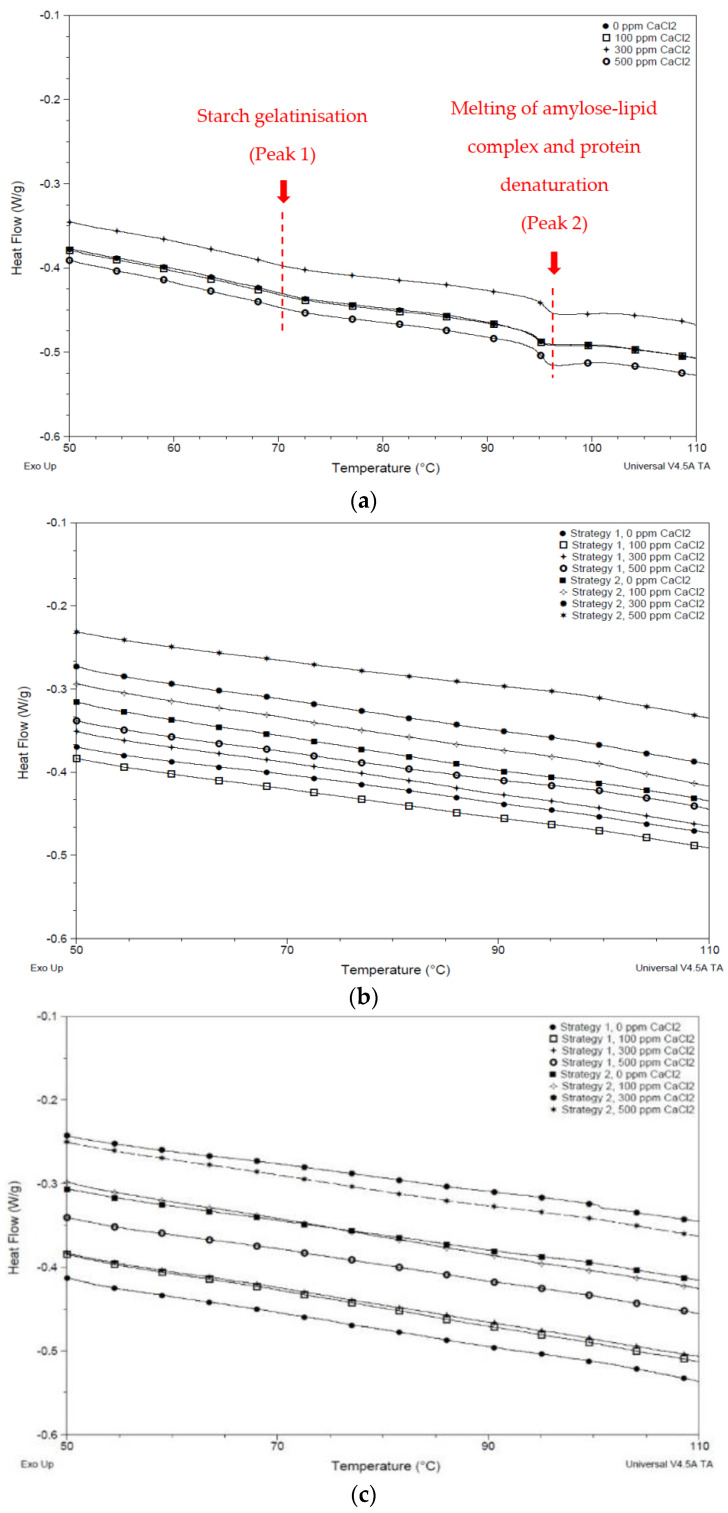
DSC thermograms of black bean flour cooked for (**a**) 0 h, (**b**) 1 h, and (**c**) 2 h in either existing soaking medium or freshly prepared CaCl_2_ solutions at 0, 100, 300, and 500 ppm.

To assess the changes in the thermal properties of black beans as a function of cooking time and CaCl_2_ treatment, the enthalpy changes of cooked bean samples using DSC were determined. Two endothermic transitions can be observed in the DSC thermograms (Figure 3), wherein the first curve was attributed to starch gelatinisation while the second one was the denaturation of protein and disruption of the amylose-lipid complex [47,58]. Starch gelatinisation usually happens before protein denaturation, as the latter occurs at higher temperatures. The formation of a lipid complex could be possible as the black bean samples, which have 2.75% fat [59], were not defatted prior to analysis in this study. The endothermic transitions with different enthalpies and temperatures can only be distinctly observed in uncooked samples (Figure 3a and Table 2). In the case of cooked (1 and 2 h) samples, there were no endothermic peaks, which suggested that a complete gelatinisation of starch, loss of amylose-lipid complex, and denaturation of protein had occurred. According to a study by Chigwedere et al. [36], starch in common beans (*Phaseolus vulgaris*) is almost completely gelatinised after 30 min of cooking. The current result also agrees with the studies of Santiago-Ramos et al. [59] and Gwala et al. [32].

The enthalpies of gelatinisation (∆*H*) for uncooked, soaked black beans at the first and second peaks were higher for CaCl_2_-treated samples (Table 2). Enthalpy indicates the energy needed to create an endothermic transition. A higher enthalpy means higher thermal energy is required to gelatinise starch, denature protein, or disrupt the lipid complexes. A significant increase in the enthalpy of starch gelatinisation was only observed at higher CaCl_2_ concentrations (300 and 500 ppm). The firming effect of calcium by forming complexes with pectin and strengthening of the middle lamella of the cell wall might have limited the diffusion of water into the starch granules which were supposed to hydrate and swell. When heat is applied, starch gelatinisation occurs, wherein the crystalline structure of starch is lost, and the amylose content leaches into the surrounding water [60]. Calcium might have restricted the swelling of starch, limiting starch gelatinisation [36].

Peak temperature (T_p_), which is the temperature when 50% of the molecules have undergone thermal transition [47], ranged from 71.00 to 72.51 °C at peak 1. It did not vary much among the samples soaked at different CaCl_2_ concentrations (Table 2). The result is in agreement with the T_p_ reported by Hoover and Ratnayake [61] for black beans, which ranged from 70 to 77 °C. The temperature range, which is the difference between the conclusion temperature (T_c_) and onset temperature (T_o_) and ranged from 7.07 to 9.40 °C, was also not affected with changing the concentration of CaCl_2_. Despite the difference in hardness levels driven by the addition of CaCl_2_, starch gelatinised at the same temperature range, although enthalpy of gelatinisation differed. A more apparent increase in T_p_ and temperature range was observed in the second peak for higher CaCl_2_ concentrations (300 and 500 ppm). A similar range was observed by Santiago-Ramos et al. [59] for raw black bean flour (94 to 105 °C). The same author had attributed the second peak to the disruption of the lipid-amylose complex in black beans, and this was observed to be present in the sample when observed with X-ray diffraction. A higher transition temperature (100–109 °C) and gelatinisation enthalpy (1.89 J/g) were also observed for black beans treated with 1.5% (*w*/*w*) CaCl_2_, employing the process called ecological nixtamalization, because CaCl_2_ has an acidic–neutral pH which is good for the environment.

This study showed that cooking of black beans (1 and 2 h), treated or untreated with CaCl_2_, completely gelatinised its starch, denatured its protein, and disrupted its lipid complex. Higher concentrations of CaCl_2_ (300 and 500 ppm) in uncooked black beans have led to an increase in enthalpy of gelatinisation.

### 3.4. The Effects of Increasing CaCl_2_ Concentration and Duration of Thermal Processing on the Starch Digestibility of Black Beans

**Figure 4:**
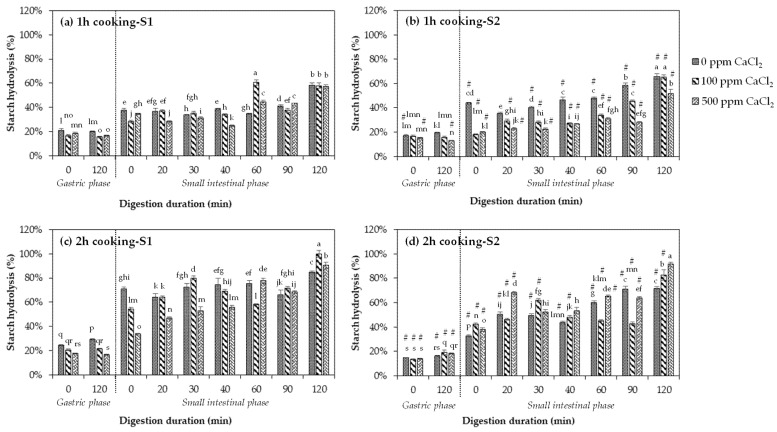
In vitro gastric and small intestinal starch digestion of black beans treated with 0, 100, and 500 ppm CaCl_2_, (**a**) cooked for 1 h under Strategy 1, (**b**) cooked for 1 h under Strategy 2, (**c**) cooked for 2 h under Strategy 1, and (**d**) cooked for 2 h under Strategy 2. Data are presented as mean ± standard deviation (*n* = 8). Starch hydrolysis values with different letters are significantly different (*p* ≤ 0.05) with increasing digestion time. Values of cooked black beans in Strategy 2 that are different from Strategy 1 at the same CaCl_2_ concentration for each digestion time point are denoted with hash (#).

Carbohydrates are the main component in legumes and are mostly composed of starch (40–50%) and dietary fibre [62]. Starch is converted to glucose by enzymes in the small intestine and digested at various degrees. Figure 4 shows the starch hydrolysis % with increasing gastric and small intestinal digestion time of black beans cooked for 1 and 2 h in different concentrations of CaCl_2_ (0, 100, and 500 ppm) using two different strategies. Uncooked black beans showed a negligible increase in digested starch and were thus excluded from the remaining discussion. This is expected as raw beans are mostly indigestible due to the presence of type 2 resistant starches (RS2). These starches are physically inaccessible due to the presence of cell walls and therefore are not readily hydrolysed by enzymes [18].

#### 3.4.1. Effects of Increasing Duration of Thermal Processing on the Starch Digestibility of Black Beans without Receiving CaCl_2_ Treatment

For black beans without CaCl_2_ treatment under both Strategies 1 and 2, there was an observed increase in starch digestibility when they were cooked for 2 compared to 1 h, as shown by the significant increase (*p* < 0.05) in starch hydrolysis % at the end of 120 min of small intestinal digestion (Figure 4, refer to 0 ppm CaCl_2_ bars). For 1 h cooked beans, starch hydrolysis was between 17% and 21% at the start of the gastric phase and reached approximately 58–66% at the end of the intestinal phase (Figure 4a,b). For 2 h cooked beans, starch hydrolysis was 15–25% at the start of the gastric phase and reached up to 71–85% at the end of the intestinal phase (Figure 4c,d). The results of the textural attributes of the black bean samples (Table 1) are useful to explain the behaviour of starch during digestion. The loss of structural integrity (i.e., decrease in hardness, cohesiveness, springiness, chewiness, and resilience) in cooked beans corresponds to an increased permeability of the cells to enzymes such as α-amylase, leading to higher starch digestion. It was found that starch hydrolysis is mostly limited by the presence of rigid cell walls in legumes and the protein matrix surrounding starch granules [32]. Thermal processing causes the protein surrounding the starch granules to denature and enables starch to undergo gelatinisation, making it accessible to amylolytic enzymes [15,62]. Due to pectin solubilisation with heat treatment, the cell wall is weakened and becomes more permeable to the diffusion of amylolytic enzymes, resulting in improved starch digestion. Numerous studies have found the same effect of cooking on starch digestibility of various legume varieties [15,62,63,64,65,66]. However, the reported values cannot be compared to other studies due to differences in the digestion methods used.

Starch hydrolysis of cooked beans improved at varying degrees for each strategy as digestion duration progressed. During 1 h of cooking, despite the similarity in textural properties of the two strategies (Table 1, 0 ppm CaCl_2_ values), black beans subjected to Strategy 2 (Figure 4b, refer to 0 ppm CaCl_2_ bars) appeared to achieve better starch hydrolysis than black beans subjected to Strategy 1 (Figure 4a, refer to 0 ppm CaCl_2_ bars), as evidenced by a higher % of digested starch in Strategy 2 (66% vs. 58% for Strategy 1) at the end of the gastrointestinal digestion. This result could be attributed to the leaching out of antinutrients such as phytic acid (soluble in water), tannin, and lectin during soaking. When not removed (i.e., Strategy 1), these can limit the digestion and absorption of starch in the gastrointestinal tract by interacting with digestive enzymes and complexing with carbohydrates. Kalpanadevi and Mohan [13] found that cooking beans without prior soaking had less of an antinutrients reduction, for example, in cowpea, only a 37% reduction was observed compared to 79% when soaking was performed before cooking. Replacing the soaking solution with a freshly prepared one (i.e., Strategy 2) would eliminate the antinutrients that had already leached out during soaking, thereby lowering the content of antinutrients in the beans and increasing the digestibility of starch. A previous study also showed that the presence of tannic acid and phytic acid reduced the digestibility of starch in legumes by up to 60% [67]. However, such finding was not observed in this study when black beans were cooked for longer (2 h) as starch hydrolysis at the end of gastrointestinal digestion was unexpectedly higher in Strategy 1 (85%) than in Strategy 2 (71%) (Figure 4c, d, refer to 0 ppm CaCl_2_ bars). The increase in starch digestibility of black beans in Strategy 1 can be due to the leaching of the simple sugars from the seeds into the medium during overnight soaking, which was not removed during cooking, possibly contributing to the high amount of hydrolysable starch measured. Several studies have reported an increase in the levels of monosaccharides, such as galactose, glucose, and fructose, disaccharides, and oligosaccharides in the soaking water of legumes including chickpeas, kidney beans, faba beans, and common beans [68,69,70,71].

This study showed that the longer black beans were cooked in absence of CaCl_2_, the more digestible their starch became. Employing Strategy 2 by replacing the soaking solution before cooking for 1 h generally further improved the hydrolysis of starch.

#### 3.4.2. Effects of CaCl_2_ Addition and Duration of Thermal Processing on the Starch Digestibility of Black Beans

Generally, the starch hydrolysis of CaCl_2_-treated black beans was found to improve with increasing cooking time (Figure 4). In other words, the application of CaCl_2_ treatment for strengthening the texture of black beans during cooking did not completely compromise the accessibility of starch towards digestive enzymes. However, increasing the CaCl_2_ concentration during cooking was found to significantly lower the extent of starch hydrolysis at certain time points along small intestinal digestion. At 1 h of cooking, CaCl_2_ treatment at 100 and 500 ppm had lowered the starch digestibility of black beans throughout the entire duration of the small intestinal digestion (Figure 4a,b). At the end of the last time point of digestion (120 min), there was no significant difference in the starch hydrolysis of the CaCl_2_-treated and untreated (0 ppm CaCl_2_) black beans cooked for 1 h in Strategy 1 (Figure 4a). A more apparent decrease in starch hydrolysis from 0 ppm CaCl_2_-treated samples after 120 min of digestion (Figure 4b) was only observed in Strategy 2 for samples cooked for 1 h in 500 ppm CaCl_2_ solution. Consistent with the texture results, a greater increase in hardness was detected in 1 h cooked CaCl_2_-treated black beans, especially in Strategy 2, with 500 ppm CaCl_2_ as the hardest. A decrease in starch hydrolysis could be desirable as it elicits a slow rise of blood glucose levels, which is helpful in managing diabetes and reduces the risk of cardiovascular diseases [11].

At 2 h of cooking, an opposite result in in vitro starch hydrolysis was observed. As shown in Figure 4c,d, starch digestibility surprisingly increased significantly (*p* < 0.05) in CaCl_2_-treated black beans cooked for 2 h employing Strategies 1 and 2 after 120 min of small intestinal digestion. Aside from interacting with pectin to strengthen the cell wall of plant tissues, calcium can also bind with phytic acid and has a catalytic effect by accelerating enzyme activity [67,72]. A study by Yoon et al. [73] revealed that the starch hydrolysis of leguminous food pressure cooked until soft increased by 40% after 5 h of in vitro digestion with the addition of calcium, which formed a complex with phytic acid. The same result was obtained by Thompson et al. [74] with navy bean bread. Furthermore, the addition of a high amount of calcium can also cause the aggregation of protein, which surrounds starch with large-pore matrices [75]. The pores enhanced the digestibility of starch by increasing the surface area and entry point of digestive enzymes, such as amylase, making it more accessible [76]. 

Comparing the two strategies of calcium uptake, starch appeared less digestible in Strategy 2 (Figure 4b,d) for almost the entire gastrointestinal digestion duration of black beans cooked for 1 and 2 h. For instance, before the start of the gastric phase, there was already a higher amount of glucose detected in Strategy 1 than in Strategy 2 for 1 and 2 h cooked black beans. Moreover, at 120 min of small intestinal digestion, black beans cooked for 2 h with 100 ppm CaCl_2_ under Strategy 1 had already completed starch hydrolysis (100%), while in Strategy 2, only 83% of starch was hydrolysed (Figure 4c,d). Starch digestibility of black beans was higher in Strategy 1, possibly due to the solubilisation of the simple sugars in the soaking water, which was not discarded during cooking as described above, thus increasing the amount of digestible starch. On the other hand, the lower starch digestibility of soaked and cooked black beans employing Strategy 2 could potentially be due to the level of calcium present in the matrix. Theoretically, there is a higher amount of calcium in freshly prepared cooking medium as the soaking solution is discarded and additional calcium is added, at the same concentration. Being negatively charged, phytic acid can chelate with cations such as calcium to form insoluble salts [77]. As phytic acid levels decrease with processing, so does calcium, especially in existing CaCl_2_ solutions. The decrease in calcium can influence starch hydrolysis due to its interaction with the pectin in the bean cell wall. This is supported by the texture result as samples in Strategy 2 were significantly harder than in Strategy 1. The strengthening of the cell walls by calcium decreased their permeability to amylolytic enzymes and limited the bio-accessibility of starch, therefore lowering the rate of starch digestion. 

The result of this study clearly showed that CaCl_2_ lowered the starch digestibility of black beans when thermally processed for 1 h. Cooking black beans for 2 h with increasing CaCl_2_ concentration improved the digestibility of starch. Cooked black beans subjected to Strategy 2 generally resulted in a lower starch digestibility than Strategy 1.

### 3.5. The Effects of Increasing CaCl_2_ Concentration and Duration of Thermal Processing on the Protein Digestibility of Black Beans

**Figure 5:**
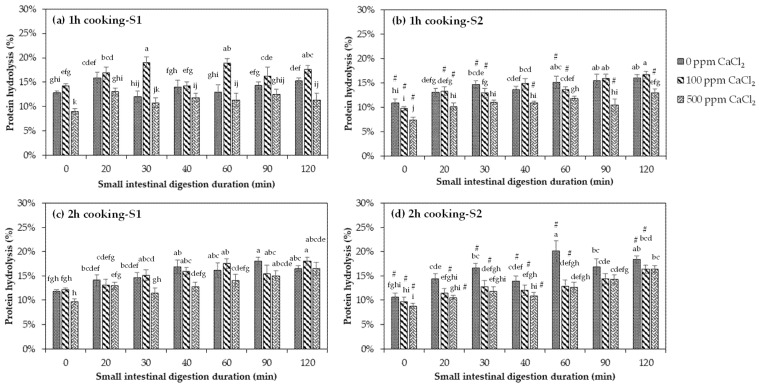
In vitro small intestinal protein digestion of black beans treated with 0, 100, and 500 ppm CaCl_2_, (**a**) cooked for 1 h under Strategy 1, (**b**) cooked for 1 h under Strategy 2, (**c**) cooked for 2 h under Strategy 1, and (**d**) cooked for 2 h under Strategy 2. Data are presented as mean ± standard deviation (*n* = 6). Protein hydrolysis values with different letters are significantly different (*p* ≤ 0.05) with increasing digestion time. Values of cooked black beans in Strategy 2 that are different from Strategy 1 at the same CaCl_2_ concentration for each digestion time point are denoted with hash (#).

Proteins are the second largest component in legumes. When ingested, their peptide bonds are hydrolysed by digestive enzymes to release small peptide chains and amino acids, which are absorbed in the body to perform their biological functions [6]. The protein digestibility of cooked and uncooked navy beans was measured using the OPA assay, wherein OPA and β-mercaptoethanol reacted with the free α-amino groups in the digests, producing 1-alkylthio-2-alkylisoindoles that has maximum absorbance at 340 nm [40]. Only the small intestinal phase is shown in Figure 5 as the amount of hydrolysed protein at gastric phase was very low (<1%) due to the minimal generation of free α-amino groups by pepsin [41]. 

#### 3.5.1. Effects of Increasing Duration of Thermal Processing on the Protein Digestibility of Black Beans without Receiving CaCl_2_ Treatment

As shown in Figure 5 (refer to 0 ppm CaCl_2_ bars), increasing the cooking duration of black beans mostly increased the amino acids and peptides released during small intestinal digestion. Protein hydrolysis after 120 min of small intestinal digestion was higher for 2 h cooked than 1 h cooked black beans, which was observed in both Strategies 1 and 2. Habiba [78] also found an increase of protein digestibility in peas with increasing cooking time. Since the storage proteins of legumes are embedded in the same cellular matrix as starch, its accessibility towards digestive enzymes was also limited by the cell wall. Cooking the beans can modulate the permeability of cell walls for enzymatic hydrolysis of protein and the subsequent absorption of its amino acids in the gastrointestinal tract [11]. The structural changes and denaturation of native protein by heat can also influence the hydrolysis of protein.

When protein is denatured, its subunits unfold and dissociate, exposing susceptible sites for enzymatic attack [79]. Carbonaro et al. [80] mainly attributed the improved digestibility of autoclaved chickpeas and common beans (by 6.23% and 9.18%, respectively) to the denaturation of protein. This was also observed by Gwala et al. [41], wherein soft legume samples (Bambara groundnuts cooked for 2 h) had higher protein denaturation and increased protein digestion. The increase of protein digestibility with thermal processing can also be due to the reduction of enzyme inhibitors, trypsin, phytic acid, and lectins [13]. Several authors have reported an increase in protein digestibility with decreasing antinutrients in certain legume species [14,16,17,66]. Thermal processing was also reported to enhance protein digestibility in horse gram, cowpeas [65,81], mung beans [82], black grams, chickpeas, lentils, and kidney beans [66]. However, these values cannot be compared in this study due to differences in the assay being used to determine protein digestibility. 

Protein hydrolysis along the 2 h small intestinal phase was consistently higher in Strategy 2 than in Strategy 1 (Figure 5c, d, refer to 0 ppm CaCl_2_ bars), despite the similarity in textural properties (Table 1, 0 ppm CaCl_2_ values), which can be due to the removal of antinutrients when the soaking solution was replaced before cooking, as explained in Section 3.4.1. Furthermore, the hydrolysis of protein in black beans was observed to be generally quite low, with the highest value obtained at 60 min of small intestinal digestion (20.11%) for samples cooked for 2 h in Strategy 2. The digestibility of protein is usually lower than starch due to the presence of globulin or phaseolin, a major storage protein in legumes, which is highly resistant to proteolysis even after cooking [83]. Furthermore, the high amount of β-sheet structure in legumes (specifically common beans), which are hydrophobic in nature, can limit the accessibility of protein digestion sites to proteases due to the formation of aggregates from protein–protein interactions during heat treatment [84]. 

This study revealed that the protein hydrolysis of black beans is generally low (≤20%). Cooking for 2 h had increased the protein digestibility of black beans but not as great as that exhibited for starch hydrolysis. Cooked black beans subjected to Strategy 2 generally resulted to improved protein hydrolysis compared to Strategy 1 in absence of CaCl_2_.

#### 3.5.2. Effects of CaCl_2_ Addition and Duration of Thermal Processing on the Protein Digestibility of Black Beans

In this study, it was found that the effect of CaCl_2_ addition on the protein digestibility of cooked beans varied with the calcium concentration applied (Figure 5). All black beans cooked in 500 ppm CaCl_2_ had a lower protein hydrolysis % compared to untreated samples (0 ppm CaCl_2_), whereas for beans treated with 100 ppm CaCl_2_, protein hydrolysis was mostly higher than those treated with 500 ppm CaCl_2_, regardless of the cooking duration and strategy adopted. For instance, at 120 min of small intestinal digestion for beans cooked for 1 h in Strategy 1 (Figure 5a), the hydrolysis of protein increased from untreated samples (0 ppm CaCl_2_) by 15.48% with 100 ppm CaCl_2_ and decreased by 25.65% with 500 ppm CaCl_2_. For black beans cooked for 1 h in Strategy 2 (Figure 5b), only those treated at 500 ppm CaCl_2_ had the lowest protein hydrolysis % at the end of small intestinal digestion. Moreover, samples cooked for 2 h in Strategy 1 (Figure 5c) shared similar protein hydrolysis % with 100 and 500 ppm CaCl_2_ after 2 h digestion at small intestinal phase. All the CaCl_2_-treated samples experienced a reduction in protein hydrolysis at all small intestinal digestion time points after cooking for 2 h in Strategy 2 (Figure 5d), with the highest decrease noted at 60 min of small intestinal digestion. However, protein hydrolysis in both concentrations (100 and 500 ppm CaCl_2_) did not differ. 

Calcium is known to crosslink with protein by binding to the free carboxylic groups of glutamic and aspartic acids (which are considerably high in black beans), forming salt bridges. The addition of a higher concentration of CaCl_2_ could have induced the aggregation of protein and calcium, which can limit the accessibility of protein to digestion due to the different localisation of amino acid residues specific for protease action [85]. This was probably the reason for the opposite effect of calcium on the digestibility of starch and protein for 2 h cooked samples with 500 ppm CaCl_2_, where protein hydrolysis decreased (Figure 5c,d) while starch hydrolysis increased (Figure 4c,d). The pores formed during the aggregation of protein and calcium might have increased the susceptibility of starch to enzymatic attack. Whether starch affects protein digestibility remains unclear, although starch did not limit the access of proteases to protein in kidney beans, as evidenced by the similar protein digestion kinetics of starch with or without α-amylase [86]. 

Increasing the cooking duration of CaCl_2_-treated black beans mostly increased the protein hydrolysis during small intestinal digestion, especially at the end of the 2 h intestinal digestion (Figure 5c,d). At most time points along the small intestinal digestion, protein digestibility improved with black beans cooked in Strategy 1 rather than in Strategy 2, which can be due to the higher level of calcium in the latter which might have strengthened the physical barrier effect of the cell wall, thus limiting the access of protein to proteases. As the proteins were still encapsulated in the cytoplasmic matrix by the cell wall even after 2 h of cooking (Figure 2), strengthening the adhesion of cells might have limited the uptake of water and protein denaturation, affecting the degree of protein hydrolysis [86]. The study by Bhattarai et al. [5] has evidenced the effect of cell wall on protein hydrolysis when the legume cells were broken, and the protein digestion was increased by almost 20 times in the absence of a physical barrier.

The protein hydrolysis values obtained in this study were lower than those reported in the literature for processed black beans [87,88] and the differences between the cooking duration and CaCl_2_ concentration applied were not substantial. This was similarly observed by Gwala et al. [41] when employing the same protein measurement method, wherein digested samples were added to TCA (to precipitate large proteins or peptides) then measured with an OPA spectrophotometric assay. Accordingly, using TCA alone might underestimate the in vivo protein digestion because not all α-amino groups are free due to the presence of small peptides. Soluble protein fragments, which are different from amino acids and peptides, are hydrolysed by pancreatic enzymes and are further digested by brush-border aminopeptidases, signalling protein digestion completion. The authors were able to obtain higher amounts of digested protein (~80% for 2 h cooked Bambara groundnuts) when additional hydrolysis was carried on the digest, which involves digesting with 6 N HCl at 100 °C for 24 h to make all α-amino groups free.

The results in this study showed that the extent of protein hydrolysis during intestinal digestion did not follow the expected linear dose–response relationship with increasing addition of CaCl_2_.

## 4. Conclusions

In this study, increasing the cooking duration of black beans, up to 2 h, was shown to severely degrade the textural properties of the beans in terms of hardness, cohesiveness, springiness, chewiness, and resilience. While light micrographs revealed that the starch granules surrounded by proteinaceous matrix were still encapsulated inside a rigid cell wall in cooked black beans, the intact parenchyma cells of black bean cotyledons did not appear to negatively influence the in vitro starch and protein digestibility of cooked beans. In fact, a greater amount of starch was being hydrolysed by digestive enzymes for those beans cooked for 2 h when compared to 1 h of cooking. Increasing concentrations of CaCl_2_ up to 500 ppm during overnight soaking and cooking was found to considerably improve the texture parameters, including hardness, cohesiveness, springiness, chewiness, and resilience, of cooked black beans. However, the impact of CaCl_2_ addition on the starch and protein digestibility of cooked beans differed according to the concentration added. The addition of a higher concentration of CaCl_2_ at 500 ppm lowered the starch digestibility of cooked black beans. It also reduced the amount of protein hydrolysed, but to a lower extent. These reductions correlated with the improved texture parameters, as the strengthening effect of calcium, when it forms crosslinks with pectin in the middle lamella of the cell wall, could have limited the accessibility of starch and protein towards digestive enzymes. Moreover, replacing the CaCl_2_ soaking solution after overnight soaking before cooking (Strategy 2) resulted in cooked black beans with increment in hardness, cohesiveness, springiness, chewiness, and resilience, and a lower starch digestibility compared to when the same CaCl_2_ soaking solution was used during cooking (Strategy 1). The ability of calcium to lower starch digestibility while still maintaining texture is a desirable characteristic. Low glycaemic index food has been an important part of the diet as it corresponds to a decrease in blood glucose response and thus is helpful in preventing and managing type 2 diabetes and cardiovascular diseases. Without calcium addition, the two strategies resulted in black beans with similar hardness, but Strategy 2 could potentially lower starch digestibility while improving the digestibility of protein, highlighting the importance of replacing the soaking solution prior to cooking.

## Figures and Tables

**Table 1:** Texture attributes of black beans soaked in different concentrations of CaCl_2_ solutions (0, 100, 300, and 500 ppm) and cooked employing two strategies for 0, 1, and 2 h.

Time of Cooking (h)	Strategy 1				Strategy 2			
	0 ppm CaCl_2_	100 ppm CaCl_2_	300 ppm CaCl_2_	500 ppm CaCl_2_	0 ppm CaCl_2_	100 ppm CaCl_2_	300 ppm CaCl_2_	500 ppm CaCl_2_
**Hardness (N)**								
0	461.67 ± 37.83 ^a^_A_	477.06 ± 37.14 ^a^_A_	481.82 ± 19.61 ^a^_A_	489.96 ± 34.78 ^a^_A_	461.67 ± 37.83 ^a^_A_	477.06 ± 37.14 ^a^_A_	481.82 ± 19.61 ^a^_A_	489.96 ± 34.78 ^a^_A_
1	31.56 ± 3.69 ^e^_B_	38.76 ± 7.32 ^de^_B_	46.82 ± 5.83 ^cd^_B_	67.00 ± 7.80 ^b^_B_	32.00 ± 5.40 ^e^_B_	48.04 ± 6.95 ^c^_B_	65.25 ± 8.14 ^b^_B_	79.29 ± 4.49 ^a^_B_
2	21.69 ± 5.98 ^def^_B_	26.05 ± 5.40 ^cde^_B_	27.09 ± 3.92 ^cd^_C_	35.34 ± 5.54 ^ab^_C_	17.17 ± 3.36 ^f^_B_	19.86 ± 2.44 ^ef^_C_	29.63 ± 2.65 ^bc^_C_	39.37 ± 7.49 ^a^_C_
**Cohesiveness (ratio)**								
0	0.26 ± 0.01 ^a^_A_	0.26 ± 0.01 ^a^_A_	0.29 ± 0.03 ^a^_A_	0.29 ± 0.03 ^a^_A_	0.26 ± 0.01 ^a^_A_	0.26 ± 0.01 ^a^_A_	0.29 ± 0.03 ^a^_A_	0.29 ± 0.03 ^a^_A_
1	0.24 ± 0.02 ^c^_A_	0.25 ± 0.01 ^bc^_A_	0.27 ± 0.03 ^ab^_A_	0.29 ± 0.02 ^a^_A_	0.25 ± 0.02 ^bc^_A_	0.26 ± 0.02 ^abc^_A_	0.26 ± 0.02 ^abc^_A_	0.27 ± 0.02 ^abc^_A_
2	0.21 ± 0.01 ^cd^_B_	0.23 ± 0.02 ^bcd^_B_	0.24 ± 0.02 ^abcd^_B_	0.25 ± 0.02 ^abc^_B_	0.21 ± 0.02 ^d^_B_	0.25 ± 0.02 ^ab^_A_	0.26 ± 0.03 ^a^_A_	0.26 ± 0.03 ^a^_A_
**Springiness (mm)**								
0	0.69 ± 0.05 ^a^_A_	0.67 ± 0.05 ^a^_A_	0.68 ± 0.06 ^a^_A_	0.74 ± 0.09 ^a^_A_	0.69 ± 0.05 ^a^_A_	0.67 ± 0.05 ^a^_A_	0.68 ± 0.06 ^a^_A_	0.74 ± 0.09 ^a^_A_
1	0.40 ± 0.04 ^c^_B_	0.41 ± 0.03 ^c^_B_	0.44 ± 0.02 ^c^_B_	0.51 ± 0.02 ^ab^_B_	0.41 ± 0.04 ^c^_B_	0.43 ± 0.03 ^c^_B_	0.49 ± 0.02 ^b^_B_	0.54 ± 0.03 ^a^_B_
2	0.31± 0.02 ^de^_C_	0.31 ± 0.04 ^de^_C_	0.35 ± 0.02 ^cd^_C_	0.41 ± 0.03 ^ab^_C_	0.31 ± 0.03 ^e^_C_	0.32 ± 0.03 ^de^_C_	0.39 ± 0.03 ^bc^_C_	0.44 ± 0.02 ^a^_C_
**Chewiness (J)**								
0	78.80 ± 4.37 ^a^_A_	84.85 ± 5.77 ^a^_A_	85.13 ± 15.83 ^a^_A_	89.16 ± 10.46 ^a^_A_	78.80 ± 4.37 ^a^_A_	84.85 ± 5.77 ^a^_A_	85.13 ± 15.83 ^a^_A_	89.16 ± 10.46 ^a^_A_
1	2.53 ± 0.59 ^d^_B_	4.05 ± 0.80 ^d^_B_	5.82 ± 0.92 ^c^_B_	9.24 ± 1.55 ^b^_B_	2.83 ± 0.71 ^d^_B_	5.74 ± 1.10 ^c^_B_	8.91 ± 1.52 ^b^_B_	12.10 ± 1.27 ^a^_B_
2	1.45 ± 0.49 ^d^_B_	2.08 ± 0.72 ^bcd^_B_	2.39 ± 0.51 ^bc^_B_	3.75 ± 0.76 ^a^_B_	1.33 ± 0.36 ^d^_B_	1.56 ± 0.35 ^cd^_C_	2.80 ± 0.56 ^b^_B_	4.43 ± 0.92 ^a^_C_
**Resilience**								
0	0.17 ± 0.02 ^a^_A_	0.17 ± 0.01 ^a^_A_	0.18 ± 0.03 ^a^_A_	0.17 ± 0.02 ^a^_A_	0.17 ± 0.02 ^a^_A_	0.17 ± 0.01 ^a^_A_	0.18 ± 0.03 ^a^_A_	0.17 ± 0.02 ^a^_A_
1	0.08 ± 0.01 ^b^_B_	0.09 ± 0.01 ^ab^_B_	0.10 ± 0.01 ^a^_B_	0.10 ± 0.01 ^a^_B_	0.09 ± 0.02 ^ab^_B_	0.10 ± 0.01 ^ab^_B_	0.10 ± 0.01 ^a^_B_	0.10 ± 0.02 ^a^_B_
2	0.07 ± 0.01 ^c^_B_	0.08 ± 0.01 ^bc^_C_	0.08 ± 0.01 ^abc^_C_	0.09 ± 0.01 ^abc^_B_	0.08 ± 0.00 ^c^_C_	0.09 ± 0.00 ^abc^_B_	0.10 ± 0.01 ^a^_B_	0.09 ± 0.01 ^ab^_B_

Data are presented as mean ± standard deviation from 10 independent measurements (*n* = 10, 5 beans per measurement resulted in a total of 50 beans). Texture values in the same row with different lowercase letters in superscript are significantly different (*p* ≤ 0.05) with increasing addition of CaCl_2_ across the two strategies for each cooking duration. Texture values in the same column with different uppercase letters in subscript are significantly different (*p* ≤ 0.05) with increasing cooking duration for each CaCl_2_ concentration.

**Table 2:** Thermal properties of uncooked black beans soaked in different concentrations of CaCl_2_. The onset (T_o_), peak (T_p_), conclusion (T_c_) temperatures, and the enthalpy for gelatinisation (∆*H*) are defined.

CaCl_2_ (ppm)	T_o_ (°C)	T_p_ (°C)	T_c_ (°C)	∆*H* (J/g)	T Range (°C)
Peak 1 (Starch gelatinisation)					
0	67.85 ^a^ (67.60–68.10)	72.14 ^a^ (71.98–72.30)	76.87 ^a^ (76.24–77.5)	0.91 ^b^ (0.90–0.92)	9.02 ^a^ (8.64–9.40)
100	65.41 ^a^ (65.31–65.50)	71.29 ^a^ (71.00–71.57)	73.76 ^b^ (73.23–74.29)	0.94 ^b^ (0.93–0.96)	8.36 ^a^ (7.92–8.79)
300	67.58 ^a^ (67.05–8.11)	71.32 ^a^ (71.07–71.56)	74.92 ^ab^ (74.34–75.50)	1.16 ^a^ (1.12–1.20)	7.34 ^a^ (7.29–7.39)
500	66.97 ^a^ (66.23–67.71)	72.41 ^a^ (72.31–72.51)	74.72 ^ab^ (74.65–74.78)	1.18 ^a^ (1.15–1.22)	7.75 ^a^ (7.07–8.42)
Peak 2 (Melting of amylose-lipid complex and protein denaturation)					
0	94.55 ^ab^ (94.40–94.70)	95.67 ^c^ (95.64–95.70)	96.59 ^b^ (96.33–96.84)	0.40 ^b^ (0.39–0.41)	2.04 ^b^ (1.93–2.14)
100	94.28 ^b^ (94.25–94.30)	95.79 ^bc^ (95.77–95.81)	96.47 ^b^ (96.41–96.53)	0.50 ^ab^ (0.44–0.57)	2.20 ^b^ (2.16–2.23)
300	95.09 ^a^ (94.97–95.20)	96.62 ^a^ (96.48–96.75)	100.65 ^a^ (100.07–101.23)	0.84 ^a^ (0.81–0.86)	5.57 ^a^ (5.10–6.03)
500	94.60 ^ab^ (94.55–94.65)	96.18 ^b^ (96.13–96.23)	98.80 ^a^ (98.51–99.08)	0.80 ^ab^ (0.68–0.93)	4.20 ^a^ (3.86–4.53)

Data are presented as mean and range in parentheses from two independent measurements (*n* = 2). Mean values with different lowercase letters in the same column for each peak are significantly different.
